# Epidemiology of *mecA*-Methicillin Resistant *Staphylococcus aureus* (MRSA) in Iran: A Systematic Review and Meta-analysis

**Published:** 2012

**Authors:** Emran Askari, Fatemeh Soleymani, Arash Arianpoor, Seyed Meghdad Tabatabai, Aminreza Amini, Mahboobeh NaderiNasab

**Affiliations:** 1 *Mashhad Medical Microbiology Student Research Group, Mashhad University of Medical Sciences, Mashhad, Iran*; 2 *MScStudent of Biostatistics, MashhadUniversity of Medical Sciences, Mashhad, Iran*; 3 *Department of Microbiology, Central Laboratory, Imam Reza Teaching Hospital, Mashhad, Iran*

**Keywords:** Iran, mecA gene, MRSA, Staphylococcus aureus, Systematic Review

## Abstract

**Objective(s):**

*Staphylococcus aureus *(*S. aureus*) is a prevalent pathogen worldwide. Methicillin resistant *S. aureus *(MRSA), which is usually multi-resistant in hospitals, has been a daunting challenge for clinicians for more than half a century. The aim of this systematic review and meta-analysis is to determine the relative frequency (R.F.) of MRSA in different regions of Iran.

**Materials and Methods:**

Search terms “S*taphylococcus aureus*”, “Methicillin”, “*mecA*” and “Iran” were used in PubMed, Scirus and Google Scholar. Two Persian scientific search engines and ten recent national congresses were also explored. Articles/abstracts, which used clinical specimens and had done PCR to detect the *mecA* gene, were included in this review. Comprehensive Meta-Analysis and Meta-Analyst software were used for statistical analysis.

**Results:**

Out of 2690 results found in the mentioned databases, 48 articles were included in the final analysis. These studies were done in Ahvaz, Falavarjan, Fasa, Gorgan, Hamedan, Isfehan, Kashan, Mashhad, Sanandaj, Shahrekord, Shiraz, Tabriz, Tehran and Tonekabon. Pooled estimation of 7464 *S. aureus* samples showed that 52.7%±4.7 (95% confidence interval [CI]) of strains were *mecA *positive. MRSA R.F. in different studies varied from 20.48% to 90% in Isfehan and Tehran, respectively. We found a moderate heterogeneity (I^2^= 48.5%) of MRSA R.F. among studies conducted in Tehran (ranging from 28.88% to 90%, mean 52.7% [95% CI: 46.6%±0.58.8%]).

**Conclusion:**

According to the results of this study, MRSA R.F. in Iran is in the high range. Thus, measures should be taken to keep the emergence and transmission of these strains to a minimum.

## Introduction

*Staphylococcus aureus* has been known as a threat to human health for more than a century. This pathogen is responsible for a wide range of maladies from folliculitis and food poisoning to life-threatening conditions such as endocarditis or necrotizing pneumonia (1).

Introduction of penicillin to the market in the 1940s was a cornerstone in treating staphylococcal infections, which was soon followed by the emergence of β-lactamase producing strains. Methicillin, a β-lactamase-resistant antimicrobial agent, was introduced in 1959. The first report of methicillin-resistant *Staphylococcus aureus* (MRSA) was from London in 1961(2-3).

It has been suggested that the *mecA* gene is responsible for resistance to methicillin. *MecA* encodes an altered penicillin-binding protein (i.e. PBP_2a_) with a low affinity for β-lactam antibiotics (2). The multi-drug resistance phenomenon, seen especially in MRSA strains, is a main cause of treatment failure and increase in treatment costs (4). It is noteworthy that MRSA infections are associated with a higher mortality rate compared to infections with methicillin-susceptible *S. aureus *(5)*.*

MRSA was previously considered as a nosocomial pathogen, but in the past two decades, reports suggest an increasing trend for community-associated MRSA (CA-MRSA). These clones may replace current health care-associated MRSA (HA-MRSA) clones in the future. This hypothesis is supported not only by mathematical models but also by reports that have shown invasion of CA-MRSA clones to hospitals (6). First described in Minnesota, CA-MRSA has now attracted global attention (1). Since 2004, MRSA related to livestock infections has also been reported. However, this type of MRSA seems to be limited to some countries, especially the ones where pig farms are common (7-8).

Recent studies have revealed an increase in the worldwide prevalence of MRSA. However, some European countries have maintained low rates of MRSA (4, 7). Although there are many reports from different cities of Iran, the average rate of MRSA in Iranian hospitals is still unknown. Our aim in this study is to provide the relative frequency (R.F.) of MRSA in Iran, as detected by the PCR amplification of the *mecA* gene.

## Materials and Methods

***Literature Search***

“*Staphylococcus aureus*”, “*S. aureus*”, “Methicillin”, “MRSA”, “MSSA”, “*mecA* gene” and Iran (for non-Iranian databases) were searched with special strategies in PubMed, Google Scholar and Scirus search engines. Two Persian scientific search engines “Scientific Information Database" (www. sid.ir), and "IranMedex" (www.iranmedex.com) were searched as well. The keywords were also searched at all Iranian academic domains (i.e. ending with.ac.ir) by “Google advanced search”. Additionally, abstract books of 10 recent congresses (i.e. “1st-5thIranian Congress of Clinical Microbiology”, “4th Congress of Laboratory and Clinic”, “First International and 12th Iranian Congress of Microbiology”, “The First Iranian International Congress of Medical Bacteriology”, “The Congress of Infections and Antibiotic Resistance” and “The Congress of Rational Usage of Antibiotics”) were explored. All common dictation mistakes and possible conditions of mentioned words (in English and Persian) were covered as well. Search strategies were followed until 17th May 2012. 

***Inclusion criteria***

Among English and Persian articles/abstracts found with above strategies, those with the following features were included in the study:

1. *S. aureus *Samples were collected from Iranian hospitals.
2. Clinical specimens were taken from patients. If there were personnel specimens as well, results of the personnel were excluded.
3. PCR method was done to detect *mecA* gene. Phenotypic results were not included because: (A) Phenotypic methods had variable sensitivities and specificities in various studies (9). (B) Phenotypic methods were affected by many factors such as pH of media, concentration of NaCl, incubation period of isolates, commercial discs and media used in different studies and also personnel’s/researcher’s skills (10). (C) Generally, avoiding heterogeneity for inclusion of studies is desirable in systematic reviews (11). (D) Breakpoints of phenotypic methods may change over time and make the interpretation of previous results more difficult. For example, Clinical Laboratory Standards Institute revised the breakpoints for cefoxitindisc diffusion and minimum inhibitory concentration in 2007 and 2008, respectively (12, 13).

***Exclusion criteria***

During observation, studies with at least one of the aspects mentioned below were excluded:

1. Samples were partially/totally selected from MRSA collections.
2. Method for detecting MRSA strains could not be discovered from the paper.

***Data collection***

At this stage, articles/abstracts with the following features were excluded as well:

1. Any projects published both in English and Persian. (In these cases, the article published later and/or with more detailed results was chosen for analysis.)
2. Duplicate publications and congress abstracts whose full-text papers were also available.
3. The origin of samples was not clear, meaning that the reviewer(s) could not find out which region or population (i.e. inpatients, personnel, or out patients) the specimens were gathered from.
4. Nasal, oral or throat swabs were taken from healthy people or patients/healthcare personnel to detect carriers.
5. Unclear report of the results, such as studies that mixed results of “Coagulase-negative Staphylococci and *S. aureus*” or “healthy people and patients”.

***Statistical analysis***

Statistical analysis was performed by the Meta-Analyst (version 3.13 Beta) and Comprehensive Meta-Analysis (version 2.0) software. Overall relative frequency of MRSA in Iran was pooled by forest plot using the Meta-Analyst software. Statistical heterogeneity of the results was checked using Cochrane Q-test with significance set at *P*< 0.1. In order to assess possible publication bias, the Begg and Mazumdar’s test was done using the Comprehensive Meta-Analysis software. The Begg and Mazumdar’s rank correlation test reports the rank correlation between the standardized effect size and the variances (or standard errors) of these effects.

## Results

**Figure 1:**
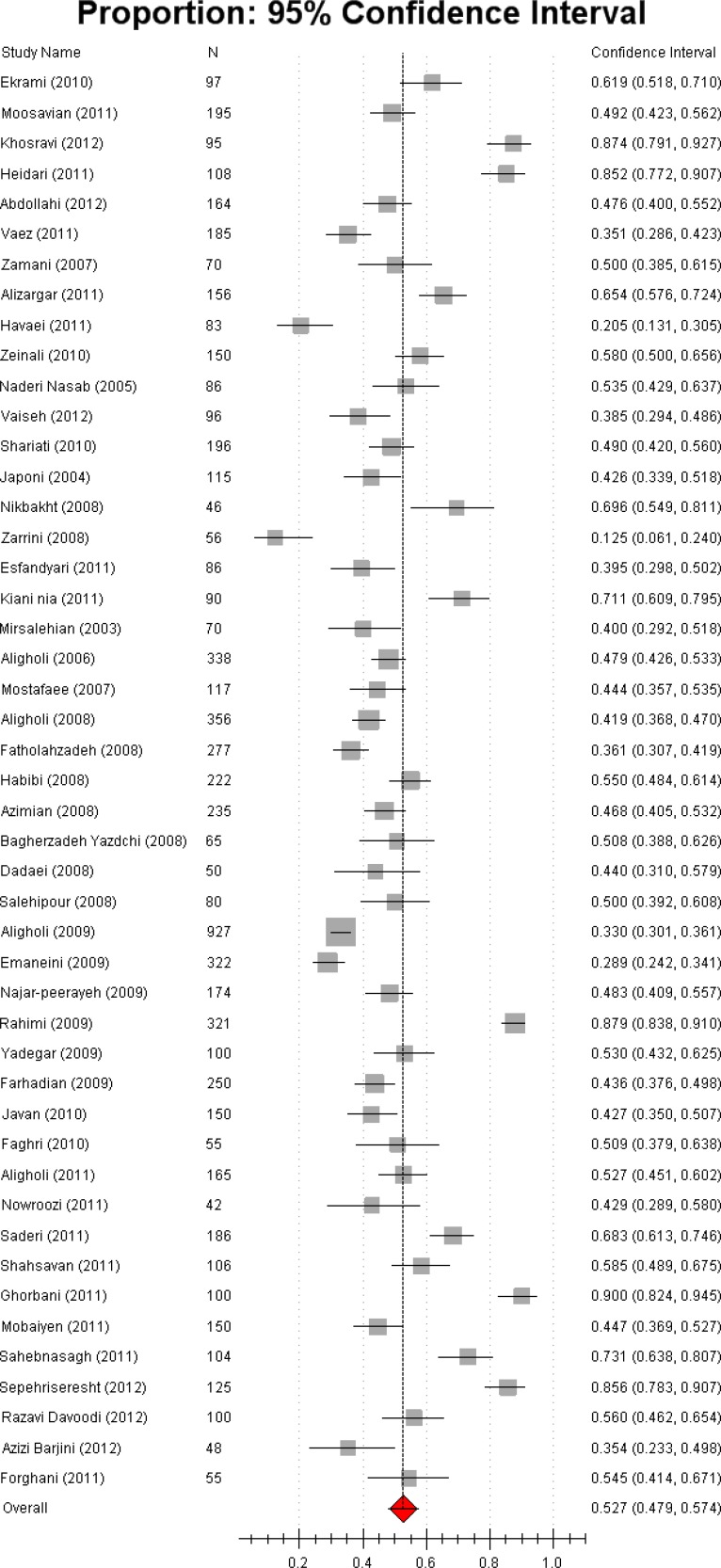
Forrest plot of the current relative frequency of *mecA*-MRSA among clinical *S. aureus* isolates in different Iranian studies

Out of 2690 articles/abstracts found by the aforementioned search strategies, 79 results matched inclusion criteria, out of which 48 (29 full-text articles and 19 abstracts) were selected for analysis (Table 1) (14-61). Sample size and 95% confidence interval (CI) of each study was shown in a forest plot (Figure 1). According to heterogeneity test, random model methods were used for meta-analysis tests (*P*< 0.001). I^2^ statistics, the proportion of variation due to heterogeneity, was 0.48, indicating moderate heterogeneity.

**Figure 2:**
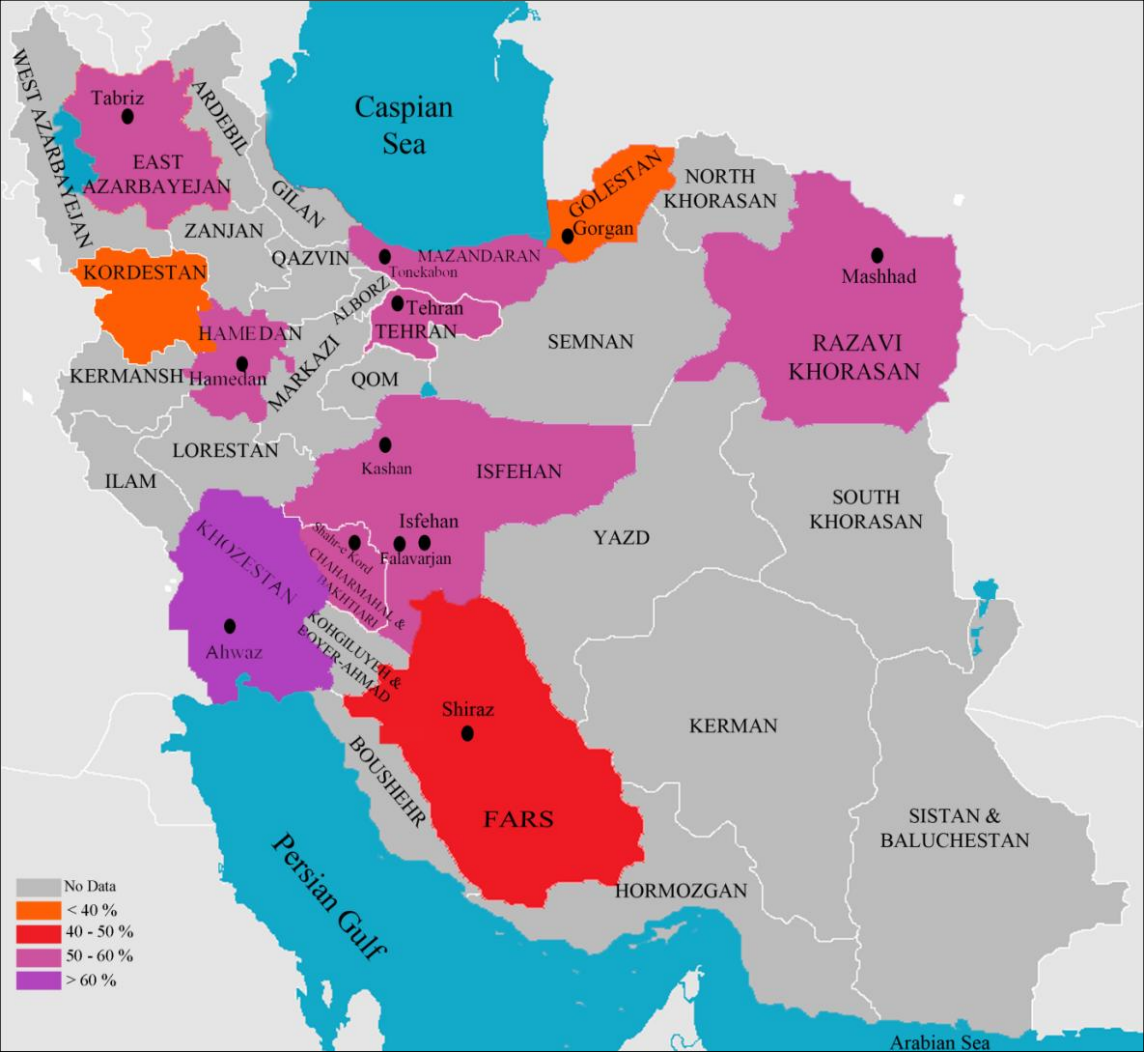
Prevalence of *mecA*-Methicillin-Resistant *Staphylococcus aureus* in Iran

Pooled estimation of 7464 *S. aureus* samples showed 52.7%±4.7 (95% CI) of strains to be *mecA *positive. These samples were taken from 14 different Iranian cities (Figure 2). MRSA R.F. varied from 20.48% to 90% in Isfehan and Tehran, respectively (22, 55). Amoderate heterogeneity (I^2^= 48.5%) of MRSA R.F. In the studies conducted in Tehran, the capital city of Iran (ranging from 28.88% to 90%, mean 52.7% [95% CI: 46.6%-0.58.8%]) (33-60) was found.

A significant correlation suggested that bias exists but does not directly address the implication of bias (Kendall’s tau= 0.21). The results of a Begg and Mazumdar’s rank correlation test supported its possibility (*P*= 0.039).

## Discussion

During the past decade, assays for detection of *mecA* gene for staphylococci became popular among Iranian researchers. Based on these studies, we reported the cumulative prevalence of MRSA and provided a map to illustrate the epidemiology of MRSA in Iran. In two previous global reports, the prevalence of MRSA in Iran was unknown (2, 7).

According to our study, the mean prevalence of MRSA in Iran was 52.7%±4.7 and was more than fifty percent in many Iranian cities. This finding indicates that physicians may face difficulties in treatment of in more than half of *S. aureus* infections. Keeping in mind the high prices of newer agents, vancomycin appears to be a suitable agent to fight this pathogen, lthough recent emergence of vancomycin resistance in Iran is really alarming (36, 62).

In a regional perspective, Iran has a higher prevalence of MRSA compared to reports from neighboring countries in the Middle East with the exception of Iraq (2, 7). The ANSORP study which reported HA-MRSA rates for eight Asian countries showed higher percentage of MRSA in those countries compared to Iran. However, judgment cannot be made because most Iranian studies did not clearly divide their *S. aureus* population to HA- and CA- infections (63).

From an international stand, our data are in the same range as Argentina and Mexico in Latin America (64). Mean Prevalence of MRSA in Iran is moderately higher than Australia and lower than the United States (65, 66). However, recent reports have shown that MRSA rates are declining in United States (67, 68). Prevalence of MRSA in Europe is heterogeneous with average lower than other continents but Portugal seems to have a similar rate of MRSA rates similar to our country (7).

**Table 1:** Sample size and MRSA strains in different studies

City	Type	Sample size	MRSA^1^	Relative frequency of MRSA (%)	Study team (Reference No.)	Year Published/ Presented
Ahvaz	Article	97	60	61	Ekrami *et al* (14)	2010
	Abstract	195	≥96	≥49.23	Moosavian *et al* (15)	2011
	Article	95	83	87.36	Khosravi *et al *(16)	2012
Falavarjan	Article	108	92	85.18	Heidari *et al *(17)	2011
Fasa	Article	164	78	47.56	Abdollahi *et al *(18)	2012
Gorgan	Article	185	65	35.13	Vaez *et al* (19)	2011
Hamedan	Article	70	35	50	Zamani *et al* (20)	2007
	Abstract	156	102	65	Alizargar *et al* (21)	2011
Isfehan	Article	83	17	20.48	Havaei *et al* (22)	2011
Kashan	Article	150	87	58	Zeinali *et al* (23)	2010
Mashhad	Article	86	46	53.48	NaderiNasab *et al* (24)	2005
Sanandaj	Abstract	96	37	38.5	Vaiseh *et al *(25)	2012
Shahrekord	Article	196	96	48.98	Shariati *et al* (26)	2010
Shiraz	Article	115	49	42.6	Japoni *et al* (27)	2004
Tabriz	Article	46	≥32	≥69.5	Nikbakht *et al* (28)	2008
	Abstract	56	≥7	≥12.5	Zarrini *et al *(29)	2008
	Abstract	86	34	39.5	Esfandyari *et al *(30)	2011
	Abstract	90	64	71	Kianinia *et al *(31-32)	2011
Tehran	Article	70	28	40	Mirsalehian *et al* (33)	2003
	Article	338	162	48	Aligholi *et al *(34)	2006
	Abstract	117	52	44.45	Mostafaee *et al* (35)	2007
	Article	356	≥149	≥41.85	Aligholi *et al* (36)	2008
	Article	277	≥100	≥36	Fatholahzadeh *et al* (37)	2008
	Article	222	122	55	Habibi *et al* (38)	2008
	Abstract	235	110	46.8	Azimian *et al* (39)	2008
	Abstract	65	≥33	≥50.8	BagherzadehYazdchi *et al* (40)	2008
	Abstract	50	22	44	Dadaei *et al* (41)	2008
	Abstract	80	40	50	Salehipour *et al* (42)	2008
	Article	927	≥306	≥33	Aligholi *et al* (43)	2009
	Article	322	93	28.88	Emaneini *et al* (44)	2009
	Article	174	≥84	≥48.2	Najar-peerayeh *et al* (45)	2009
	Article	321	282	88	Rahimi *et al* (46)	2009
	Article	100	53	53	Yadegar *et al* (47)	2009
	Abstract	250	109	≥43.6	Farhadian *et al* (48)	2009
	Article	150	64	≥42.67	Javan *et al* (49)	2010
	Abstract	55	28	50.9	Faghri *et al* (50)	2010
	Article	165	≥87	≥52.72	Aligholi *et al* (51)	2011
	Article	42	18	42.8	Nowroozi *et al* (52)	2011
	Article	186	127	68.3	Saderi *et al* (53)	2011
	Article	106	62	58.49	Shahsavan *et al* (54)	2011
	Abstract	100	90	90	Ghorbani *et al* (55)	2011
	Abstract	150	67	44.6	Mobaiyen *et al* (56)	2011
	Abstract	104	76	73.1	Sahebnasagh *et al* (57)	2011
	Article	125	107	85.6	Sepehriseresht *et al *(58)	2012
	Article	100	56	56	RazaviDavoodi *et al *(59)	2012
	Abstract	48	17	35.4	AziziBarjini *et al *(60)	2012
Tonekabon	Abstract	55	30	≥54.54	Forghani *et al* (61)	2011

^1^ MRSA strains were detected/confirmed by PCR amplificationof*mecA*gene

PCR of *mecA*was done onlyfor strains resistant to methicillin by phenotypic methods

Results were obtained by comparing references (31) and (32)

The heterogeneity of MRSA prevalence at national and international level is not completely understood. Possible explanations are different in infection control practices, antimicrobial administration, human population, predominant strain(s), study design and laboratory testing for determining resistance (2, 69).

This study has some limitations. First, it cannot fully represent Iran because there were no data on *mecA*-MRSA from many parts of the country. However, as described above, this is preferred to mixing the results from different phenotypic methods with genotypic ones. Second, due to limited access to in-press articles and theses, some studies might have been missed, which is also suggested by statistical analysis.

## Conclusions

Our study showed that the mean MRSA R.F. among Iranian studies is in the high range. Thus, measures should be taken to keep the emergence and transmission of these strains to a minimum.
